# S16-3 Hungarian Sporting Nation Programme

**DOI:** 10.1093/eurpub/ckae114.272

**Published:** 2024-09-26

**Authors:** Gregor Jurak

**Affiliations:** University of Ljubljana, Faculty of Sport, Slovenia

## Abstract

**Purpose:**

The purpose of the Sporting Nation Programme is to ensure access to everyday physical activity and exercise for the Hungarian population. It is an important goal, that parallel with being a successful nation in professional sports, the Hungarian nation should be also a sporting nation at community and grassroots level. The mission is to make physical activity and sport a lifelong regular routine.

**Project or policy description:**

The Sporting Nation Programme had been launched in September 2023, as an overarching initiative of the Hungarian sport governance. The State Secretariat for Sport has initiated the programme and the National Sport Agency is in charge of implementing it. The focus is on ensuring opportunities and provide the necessary conditions for people to access daily physical activity and sporting services. The programme has the following elements:

- cooperation with sport federations, clubs, coaches: aiming to open sport clubs for the wider public, not only providing sporting opportunity for the professional athletes but also for everyday people

- Sporting Nation Club: a club offering discounts to different sport services. Members of the Sporting Nation Club have a membership card, that enables the card holders to receive discounts from entry fees to sport facilities maintained by the state and to different sport events.

- physical activity on the spot of major sport events: in the framework of the programme, top sport events will be accompanied with recreational sport opportunities for fans, spectators and club members.

Dissemination of the programme is continuously happening under the coordination of the National Sport Agency. More and more clubs, venues and events become part of the programme. There is also thematic planning, in 2024 the public street workout parks and gyms will be promoted within the programme with the support of the QR code system that offers a tailored training program for users.

**Conclusions:**

The State Secretariat for Sport together with the National Sport Agency are continuously developing and monitoring the programme with the aim to find the best offers that successfully engage citizens. The programme coordinators look for partnerships to strengthen the reach-out capacity of the programme, thus in 2024 the period of the European Week of Sport will be important.

